# Inhibiting lysosomal aldolase: a magic bullet for AMPK activation in treating metabolic disease?

**DOI:** 10.1093/lifemeta/loac027

**Published:** 2022-10-10

**Authors:** David Carling

**Affiliations:** MRC London Institute of Medical Sciences, Imperial College, London W12 0NN, UK

**In a tour-de-force study by Zhang *et al.* recently published in *Nature Metabolism*, a newly identified aldolase inhibitor, Aldometanib, is shown to activate lysosomal AMP-activated protein kinase (AMPK). Remarkably, mice treated with Aldometanib have increased insulin sensitivity, lowered blood glucose, and decreased hepatic steatosis and fibrosis, and are long-lived, effects of which all appear to be mediated via activation of lysosomal AMPK.**

AMPK is a central regulator of mammalian energy homeostasis, and activation of AMPK regulates a wide range of biological processes that are linked to multiple health benefits, associated with an increased healthy lifespan [1]. Designing efficacious AMPK activators for clinical use that show long-term safety profiles has proved challenging, but the potential reward for such drugs would be substantial. The new study by Zhang *et al*. [2] builds on previous work from the same authors that identified a pathway through which glucose limitation leads to activation of AMPK, independent of changes in adenine nucleotides [3, 4]. Classically, activation of AMPK usually occurs in response to a fall in cellular ATP, which gives rise to an increase in the levels of ADP and AMP. Once activated, AMPK switches-off ATP-consuming anabolic pathways and switches-on ATP-generating catabolic pathways, helping to restore ATP levels [1]. In a series of pioneering studies, the group led by Shengcai Lin revealed a mechanism whereby glucose starvation activates AMPK without an increase in AMP or ADP levels [3−5]. In this nucleotide-independent activation pathway, decreased fructose 1,6 bisphosphate (FBP) in response to glucose limitation plays a crucial role.

Aldolase catalyses the reversible conversion of FBP into dihydroxyacetone phosphate and glyceraldehyde-3-phosphate. Binding of FBP to aldolase localized to the lysosomal membrane through its interaction with the lysosomal proton pump vacuolar H^+^-ATPase (v-ATPase) was shown to regulate the interaction of AMPK with its upstream kinase, liver kinase B1 (LKB1), thereby altering phosphorylation and activation of lysosomal AMPK [3−5]. Whilst this mechanism involves a number of other proteins, the overall effect is that following glucose limitation, a decrease in FBP concentration means that aldolase is no longer bound to FBP, and the interaction between LKB1 and AMPK is promoted, resulting in phosphorylation and activation of AMPK [3−5]. An important feature of this mechanism is that activation of AMPK is restricted to the lysosomal pool of AMPK, leaving other pools of AMPK (e.g. cytosolic, mitochondrial, and nuclear) unaffected [6].

In their latest study, Zhang *et al.* hypothesized that molecules that compete for FBP binding to aldolase would mimic glucose starvation and thereby lead to activation of AMPK. Moreover, the authors speculated that this activation would be limited to the lysosomal pool of AMPK and might therefore have some advantages over more widespread AMPK activation seen with direct pharmacological agonists. Using an assay to identify inhibitors of aldolase, LXY-05-029, which the authors refer to as Aldometanib, was identified. In cell-free assays, Aldometanib inhibited aldolase with an IC_50_ of ~50 μmol/L.

Consistent with this, binding studies indicated a dissociation constant (K_D_) of around 20 μmol/L. Despite this relatively weak binding, the authors went on to investigate whether treatment of cells with Aldometanib affected AMPK activity. Aldometanib had no detectable effect on glycolytic rate or on adenine nucleotide levels for concentrations <200 nmol/L, but concentrations as low as 5 nmol/L caused a significant activation of AMPK in a range of mammalian cells. These results are surprising, not least because AMPK activation is seen with a concentration of Aldometanib ~10,000-fold lower than the IC_50_ value reported using purified aldolase in the cell-free assays. Compounding this apparent disconnect, FBP levels in glucose fed cells is present at high micromolar levels, and would presumably compete for binding of Aldometanib, increasing further the IC_50_ value for aldolase in cells. How might these differences in IC_50_ between cells and cell-free data be explained? The authors show that Aldometanib accumulates in the lysosomal fraction, possibly due to the low pH of the lysosome. However, this affect alone is unlikely to account entirely for the difference, and so another possibility is that, as yet unknown, other factors present in the lysosomal environment increase the affinity of aldolase for Aldometanib. This possibility is supported by experiments showing that aldolase present in lysosomal fractions isolated from mouse embryonic fibroblasts (MEFs) is inhibited by Aldometanib with an IC_50_ of ~15 nmol/L, >3000-fold lower than the IC_50_ determined using purified aldolase. Identifying the nature of these additional factors present in the lysosomal fraction will be an important goal in future studies.

Armed with their new aldolase inhibitor, the authors set about exploring the effect of Aldometanib *in vivo*. Consistent with the finding that Aldometanib treatment activates AMPK in cells, the authors found that Aldometanib leads to a number of beneficial metabolic effects that overlap closely with previous results obtained using direct AMPK activators [7, 8], or a genetic gain-of-function AMPK mouse model [9]. A striking feature of the effects of Aldometanib treatment is the finding that it appears to phenocopy all of the beneficial metabolic effects that have been reported for AMPK activation, including improved glucose homeostasis through increased glucose uptake into skeletal muscle, reduced hepatic steatosis, protection against nonalcoholic steatohepatitis, and a marked reduction in diet-induced obesity by reducing fat mass. Adding to these metabolic effects, the authors showed that Aldometanib extends lifespan in *Caenorhabditis elegans* (*C. elegans*), supporting previous studies linking AMPK activation to increased longevity in worms [10]. Extending their studies to mice, the authors showed that 1 year old mice treated with Aldometanib for 4 months have improved exercise performance. Finally, in a ‘coup de grace’, the authors reported that mice treated with Aldometanib from 1 year of age show a modest increase in lifespan.

So, does this mean that Aldometanib is a new ‘wonder drug’? One of the safety concerns surrounding AMPK activation is that gain-of-function mutations in the *PRKAG2* gene (coding for the γ2 subunit of AMPK) lead to a cluster of severe cardiac abnormalities [1]. Importantly, in the current study, mice treated with Aldometanib showed no adverse cardiac effects. One possibility for the lack of adverse cardiac effects is that AMPK activation by Aldometanib appears to be restricted to the lysosomal pool of the kinase. If this is the case, it would imply that all of the beneficial effects of Aldometanib are mediated by activation of lysosomal AMPK. At first glance, this seems unlikely as it would point towards a redundant role for the majority of cellular AMPK. However, it is possible that chronic activation of a small pool of AMPK is capable of compensating for transient activation of ‘bulk’ AMPK. One major disease that was not covered by the study was cancer. The role of AMPK in cancer remains controversial with studies supporting both a tumour-suppressor and tumour-promoter role, and there are a number of situations where activation of AMPK may be detrimental [1]. Determining the effect of Aldometanib in cancer disease progression represents an important challenge.

**Figure 1:**
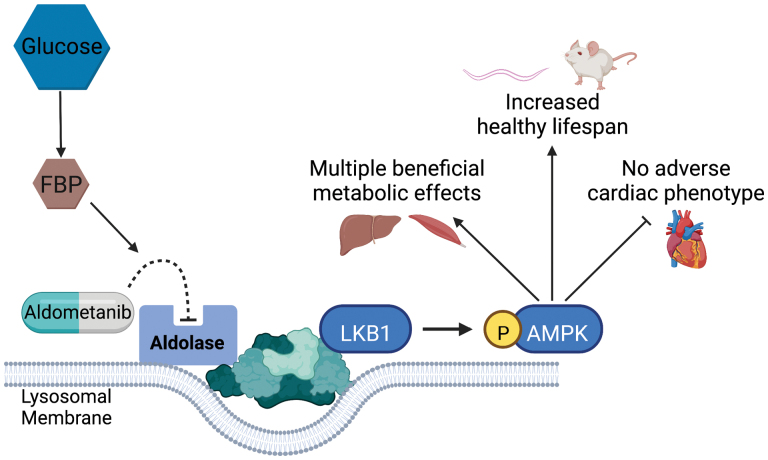
Aldometanib mimics glucose starvation leading to activation of AMPK. Aldometanib competes for binding of FBP to aldolase at the lysosomal membrane. FBP-unbound aldolase initiates a series of events that ultimately leads to phosphorylation and activation of AMPK by LKB1. This mechanism involves a number of proteins at the lysosomal membrane including v-ATPase, the calcium channel transient receptor potential V and axin, as well as other, as yet, uncharacterized factors. Although the mechanism remains enigmatic, the localization of aldolase at the lysosomal membrane greatly lowers the IC_50_ for Aldometanib and results in activation of lysosomally localized AMPK. Once activated, AMPK induces a number of beneficial metabolic effects, including increased glucose uptake into skeletal muscle, reduced hepatic steatosis and fibrosis, and protection against diet-induced obesity. Moreover, chronic treatment with Aldometanib increases lifespan in *C. elegans* and mice, with no adverse cardiac phenotypes detected in mice.

However, the current study provides further compelling support that pharmacological activation of AMPK provides an attractive target for preventing and treating metabolic disease. Inhibiting lysosomal aldolase provides another avenue for achieving this aim, and may prove to be a long sought after magic bullet (Fig. 1).
